# Harming patients by provision of intensive care treatment: is it right to provide time-limited trials of intensive care to patients with a low chance of survival?

**DOI:** 10.1007/s11019-020-09994-9

**Published:** 2021-01-15

**Authors:** Thomas M. Donaldson

**Affiliations:** University of Manchester, Oxford Road, Manchester, M13 9PL UK

**Keywords:** Intensive care, Dying, Medical decision-making, Time-limited trials

## Abstract

Time-limited trials of intensive care have arisen in response to the increasing demand for intensive care treatment for patients with a low chance of surviving their critical illness, and the clinical uncertainty inherent in intensive care decision-making. Intensive care treatment is reported by most patients to be a significantly unpleasant experience. Therefore, patients who do not survive intensive care treatment are exposed to a negative dying experience. Time-limited trials of intensive care treatment in patients with a low chance of surviving have both a small chance of benefiting this patient group and a high chance of harming them by depriving them of a good death. A ‘rule of rescue’ for the critically unwell does not justify time-limiting a trial of intensive care treatment and overlooks the experiential costs that intensive care patients face. Offering time-limited trials of intensive care to all patients, regardless of their chance of survival, overlooks the responsibility of resource-limited intensive care clinicians for suffering caused by their actions. A patient-specific risk–benefit analysis is vital when deciding whether to offer intensive care treatment, to ensure that time-limited trials of intensive care are not undertaken for patients who have a much higher chance of being harmed, rather than benefited by the treatment. The virtue ethics concept of human flourishing has the potential to offer additional ethical guidance to resource-limited clinicians facing these complex decisions, involving the balancing of a quantifiable survival benefit against the qualitative suffering that intensive care treatment may cause.

## Time-limited trials of intensive care

The ageing population of many countries has led to more elderly and increasingly co-morbid patients accessing healthcare services, and intensive care is no exception. There is an increasing proportion of elderly patients on intensive care units (Heyland et al. 2015; Fuchs et al. 2012), and both age and co-morbidity are predictors of mortality at 1 year (Gayat et al. 2018). At 1 year, only 26% of patients aged 80 and above have achieved a recovery to baseline function (Heyland et al. 2015) and the 1-year mortality of ICU patients older than 85 years is 56%, compared to 36% for patients aged 65 to 75 years (Fuchs et al. 2012). Poor outcomes from intensive care treatment have been linked to patients with more than one co-morbid condition (Esper and Martin 2011), and co-morbidity is common in elderly patients (de Rooji et al. 2005). This creates an increasing demand for intensive care treatment for patients with a low chance of surviving their critical illness, even with intensive care treatment. Time-limited trials of intensive care have arisen in response to this.

The practice of a time-limited trial of intensive care is the provision of life-sustaining treatment to a patient for a pre-determined duration, with an assessment of their response to the treatment at the end (Vink et al. 2018). Continuation of treatment is dependent upon a demonstratable improvement in the patient’s condition. Trials of intensive care are offered to patients suffering from underlying conditions with a poor prognosis (Shrime et al. 2016) and a limited life expectancy, even before they became critically unwell (Quill and Holloway 2011). They have even been suggested for patients with as little as a 5% chance of survival with intensive care treatment (Quill and Holloway 2011). Trials of intensive care have developed as a response to the clinical uncertainty inherent in decision-making for critically ill patients. The prediction of which patients will survive their critical illness with intensive care treatment has proved unreliable (Detsky et al. 2017), and the desire to ensure that no-one who may survive is denied intensive care treatment has led to calls for trials of increasing duration (beyond 15 days) (Shrime et al. 2016), despite the acknowledged risk of patient discomfort (Quill and Holloway 2011). This approach could lead to a clinical practice where every critically unwell patient, no matter how low their chance of survival, is given a time-limited trial of intensive care treatment. However, it is important to consider the potential harms that this clinical practice could cause.

## Survivors experiences highlight the harms of intensive care treatment

A sedated intensive care patient receiving mechanical ventilation may have the appearance of being unconscious, but follow-up studies with survivors show that many patients have memories of their experiences, which have been described as frightening and chaotic, leading to feelings of instability, vulnerability and fear (Samuelson et al. 2007). These stressful experiences have led to calls for increasing the depth of sedation of intensive care patients (Samuelson et al. 2007). However, deep sedation results in an increased incidence of patients suffering from delusional memories, hallucinations and nightmares, causing panic, anxiety and confusion (Samuelson et al. 2006).

The incidence of recall in survivors of intensive care varies across studies from 36.3 to 95% (Merilainen et al. 2013; Rundshagen et al. 2002). Consequently, there remains a degree of uncertainty as to the scale of the problem. Studies have reported recall of pain (52%), disturbing dreams (21–73%), procedures (69%), thirst (62–76%), noise (38–49%), difficulty communicating (65%), difficulty swallowing (44%) and awareness of invasive tubes (17%) (Rundshagen et al. 2002; Rotondi et al. 2002; Alasad et al. 2015). Studies also reported various emotional experiences such as panic/fear (32–41%), helplessness (44%), lack of control (46%), feeling tense (46%) and patients thinking they were dying (36–38%) (Rundshagen et al. 2002; Rotondi et al. 2002; Alasad et al. 2015).

These negative and stressful experiences can lead to symptoms of post-traumatic stress disorder in survivors of intensive care treatment (Davydow et al. 2013). Despite this, 86.5% of survivors of intensive care treatment retrospectively agree with the decision that was made to mechanically ventilate them (Mendelsohn et al. 2002), and 75.9% of survivors would choose to go through mechanical ventilation again (Guenter et al. 2006). However, it is not possible to ask patients who did not survive whether they retrospectively agree with the decision to provide them with intensive care treatment.

It must be assumed that all intensive care patients (survivors and non-survivors) have similar experiences of their treatment. Therefore, the negative experiences described by survivors must be assumed to be shared by patients who die whilst receiving such treatment. Consequently, dying whilst receiving intensive care treatment is likely to be a significantly distressing experience. A direct comparison of the dying experiences of intensive care patients and palliative care patients is clearly not possible, because no one can report back after they have died. However, it is likely that dying whilst receiving analgesic symptom control, in a quiet environment surrounded by loved ones is preferable to dying whilst receiving intensive care treatment. This means that the provision of intensive care treatment has the potential to cause significant harm to patients who do not survive their critical illness, by depriving these patients of a better dying experience that might otherwise have been provided for them by palliative care.

## Is it right to provide time-limited trials of intensive care to patients with a very low chance of surviving their critical illness?

There are several pressures upon intensive care clinicians which could lead them to offer time-limited trials of intensive care treatment to critically unwell patients with a very low chance of survival. The threat of legal action and pressure from a patient’s family or other clinicians can influence a clinician to inappropriately admit a patient to an intensive care unit (Giannini and Consonni 2005). These external pressures can easily become the dominant force driving intensive care decision-making. Therefore, an intensive care patient who cannot make their perspective known is in a particularly vulnerable position. A relative can bring a legal action if they think an intensive care clinician failed to prevent a critically unwell patient from dying. However, a non-survivor of intensive care treatment will never be able to seek legal redress if they were harmed by an intensive care clinician who caused them to be exposed to a negative dying experience.

The vulnerability of the critically unwell patient and the complexity of the clinical decision-making mean that clear ethical guidance is needed to help clinicians decide the right course of action. Deontological (rule based), consequentialist (outcome based) and virtue (character based) ethics approaches are widely used to help guide medical ethical decision making. This paper will examine whether these ethical approaches support the use of time-limited trials of intensive care for critically unwell patients with a very low chance of survival.

## Rule-based ethics: is there a ‘rule of rescue’ to critically unwell patients regardless of the cost?

The ‘rule of rescue’ has been defined as “the injunction to rescue identifiable individuals in immediate peril regardless of cost” (Honeybul et al. 2011). This is applicable to critically unwell patients who will die unless rescued by intensive care treatment and is an example of a deontological ethical approach. Deontological theories emphasise the duty to follow moral rules, regardless of the likely outcome of the resulting action (Garbutt and Davies. 2010).

The uncertainty inherent in prognostication means that the only way to ensure that no preventable deaths occur would be to provide all critically unwell patients with a trial of intensive care treatment. If the paramount ethical concern for intensive care clinicians is to avoid any preventable deaths from occurring, then this creates an ethical duty to ensure that no critically unwell patient dies without a trial of intensive care treatment. This would, in effect, be a ‘rule of rescue’ and has been shown to be an important factor in intensive care decision-making (Kohn et al. 2011).

The costs referred to in the definition of the rule of rescue are usually considered as opportunity costs, where the resources could have been utilised to greater benefit elsewhere in society. The importance of resource limitation has been brought into sharp focus by the COVID-19 pandemic, when sufficient resources may not exist to discharge a ‘rule of rescue’ duty for all the patients in need of intensive care treatment (Wilkinson 2020). However, the experiences of intensive care patients demonstrate that the costs of intensive care treatment are not only borne by society. There are significant costs in terms of the suffering that intensive care patients face, including the cost of a negative dying experience. Whilst a life-saving rescue from critical illness may justify a degree of suffering to patients, the rule of rescue requires intervention “regardless of cost”. Such a formulation could be used to justify inflicting any amount of suffering on an individual for even the slightest chance of survival. Yet, for any chance of survival there will be a degree of suffering that is unacceptable to a patient and this represents their own resource limitation regarding the costs of intensive care treatment. The experiences and perspectives of intensive care patients are vitally important to assess the reality of the suffering caused by intensive care treatment and to ascertain what level of suffering patients are willing and able to bear, for any given chance of survival.

A ‘rule of rescue’ regarding the provision of intensive care treatment may also have implications for when a trial of treatment can be ended. There is no logical reason why an obligation to prevent the death of a critically unwell patient should cease to apply after a certain period of time. This would indicate that intensive care treatment should only be ended when there is absolute certainty that no recovery is possible for the patient, rather than after a pre-determined period of time during a time-limited trial of intensive care treatment.

Therefore, whilst a ‘rule of rescue’ regarding critically unwell patients describes an ethical imperative to strive to maximise the survival of patients, there are logical problems using such an approach as an ethical basis for time-limited trials of intensive care. A ‘rule of rescue’ applied regardless of cost would result in significant harms for patients with a low chance of survival. To prevent well-meaning rescue attempts causing significant harm to critically unwell patients, these costs must be considered and carefully weighed against the potential for benefit.

## Outcome based ethics: the need to balance quantitative and qualitative harms

Consequentialist ethical approaches assess the rightness of a course of action with reference to the outcome(s) of the action (Savulescu and Wilkinson 2019). The most influential consequentialist theory is utilitarianism which defines right action as the action which maximises happiness (Singer 2003). Whilst the difficulties quantifying happiness limit the clinical use of utilitarian calculus, a risk–benefit analysis is an important outcome-based practical decision-making tool that is widely employed in clinical practice (Beauchamp and Childress 2009).

An ethical obligation to provide all critically unwell patients with time-limited trials of intensive care could be derived from a consequentialist imperative to achieve survival as the optimal outcome for patients. This would create a negative responsibility (Harris 1985) upon intensive care clinicians for the death of any critically unwell patient that might have been saved by intensive care treatment. Such a responsibility can be implied by families and other doctors, who put pressure on intensive care clinicians to inappropriately admit critically unwell patients with a low chance of survival to intensive care units (Giannini and Consonni 2005).

However, negative responsibility to prevent harm from occurring creates an obligation on moral agents to spend all of their time, resources and bodily energy to ensure the best possible outcome, with no freedom to decide which goods to pursue as the focus of their life and endeavours (Williams and Smart 1973). Human moral agents have a limited resource of time and a finite number of other resources (e.g. ventilators) at their disposal. Most fundamentally, a human being has their body, which is a non-transferable requirement for life and the primary means by which agents execute their agency in the world (Woollard 2013). As a resource-limited finite human, an agent must make choices, limiting their endeavours to certain goods, so leaving other goods undone. An agent cannot be held responsible for every harm that they could possibly have prevented, as they lack the resources to achieve this. However, when an agent deploys some of their limited resources to generate certain outcomes, this choice is the reason that responsibility can be ascribed for the outcome that they have caused.

The importance of resource limitation in intensive care has been highlighted by the current COVID-19 pandemic (Wilkinson 2020). As resource-limited moral agents, intensive care clinicians bear responsibility for the harms that result from their actions, and as such they should decide when the survival benefit of intensive care treatment justifies exposing the patient to the risk of suffering. They should also decide when to withhold intensive care treatment to avoid causing more harm than good to a patient. The provision of time-limited trials of intensive care treatment to all critically unwell patients, even those with very low chances of surviving their critical illness, overlooks the harms that intensive care treatment can cause. This may cause clinicians to lose focus on their responsibility for the experiential harms that result from their decisions to employ their limited resources. As resource-limited moral agents, intensive care clinicians should ensure that they do not use their limited intensive care resources in ways that cause unjustifiable suffering to their patients.

The possibility of exposing patients to an unnecessarily distressing dying experience should, therefore, be considered in a consequentialist ethical analysis of intensive care decision-making. The suffering inherent in intensive care treatment and the chance of exposing the patient to a negative dying experience must be carefully weighed against the chance of survival. Therefore, a patient-specific risk–benefit analysis is an important component of intensive care decision-making.

The intrinsic difference in importance between survival and a positive experience of dying may justify some weighting of the risk–benefit analysis in favour of survival benefit. However, co-morbidity, age and limited life expectancy associated with pre-existing conditions with a poor prognosis, also predict a lower quality of life and reduced physical function after surviving a critical illness (Dowdy et al. 2005). Therefore, patients who have a very low chance of benefitting by surviving their critical illness, also face a reduced benefit in terms of potential quality of life if they do survive, as well as the increased likelihood of a negative dying experience.

When undertaking a risk–benefit analysis, the harms of intensive care have the potential to be underestimated or even go unrecognised. In particular, the possibility of causing a negative dying experience can easily be a hidden harm. One reason for this is simply that patients are unable to report back with their perspectives on their dying experience. Furthermore, survival benefit can be quantitatively measured, whereas an experience of dying is by nature subjective. This may lead advocates of time-limited trials of intensive care to focus on avoiding the quantifiable harm of failing to prevent an avoidable death, whilst overlooking the qualitative human suffering involved.

Consequentialism highlights the importance of a risk–benefit analysis when deciding whether to offer a critically unwell patient a time-limited trial of intensive care treatment. However, clinicians face the practical difficulties of applying these theories in a complex scenario involving both uncertainty as well as the need to consider both quantitative and qualitative outcomes. There is no clear ethical guidance available to clinicians seeking to weigh between quantitative and qualitative outcomes in a risk–benefit analysis. Without further resources and guidance, clinicians in the current environment of evidence-based medicine can easily overlook qualitative outcomes. By focussing on the quantifiable outcome of survival to the exclusion of qualitative experiential outcomes, advocating time-limited trials of intensive care for all critically unwell patients, regardless of chance of survival, has the potential to result in the harm of negative dying experiences for increasing numbers of patients. Therefore, whilst a patient-specific risk–benefit analysis is an important component of clinical decision making, further ethical resources are required to support clinicians in deciding whether to provide a patient with intensive care treatment.

## Virtue ethics: accepting complexity and human limitation

Clinical decision-making regarding the provision of intensive care treatment to critically ill patients is, by necessity, complex and involves multiple considerations, including clinical factors and the patient’s wishes. Critical illness forces the patient to face the starkest of human limitations, that of the finiteness of their life. However, critical illness also forces the intensive care clinician to face their own limitations as a moral agent. These limitations may manifest in terms of a lack of life-saving intensive care resources for all the patients who need them, as well as in limitations in prognostic accuracy and inability to ensure a good outcome for the patient even with best treatment. The complexity of the decision to be made, combined with the human limitations of both the patient and the doctor create a scenario which requires more guidance than is offered by traditional deontological or consequential approaches.

A potential avenue for further ethical analysis of this issue is virtue ethics, which places key emphasis on the character of the moral agent, including their rationality, emotions and motivation, whilst encouraging flexibility and creativity to adapt to different situations and circumstances (Gardiner 2003). Virtue ethics seeks to ground its account of the virtues in the context of human experience (Chappell 2015), and mortality and the resource-limitation inherent in being an embodied creature are essential elements of the human experience (Campbell 2003).

Virtue ethics seeks to provide is an account of human flourishing (eudaimonia) which acts as a goal for virtuous action and, therefore, a guide for ethical decision-making (Aristotle 2009, 1098a15). This concept is much more than simple happiness, but instead considers flourishing in terms of a complete human life, which may not be fully assessable until that life is ended (Aristotle 2009, 1100a20). Therefore, death is included in the virtue ethics concept of human flourishing and dying well is an important part of flourishing as a human.

Whilst this appears to be a promising approach to the complexity of intensive care decision-making, the current lack of a consensus regarding the key virtues is a major limitation for its application in clinical decision-making (Veatch 1985). Virtue ethics can also be criticised for requiring the agent to balance the demands of multiple virtues, which may well be contradictory. In a context such as intensive care decision-making this could be seen to add confusion into an already complex situation. However, an ethical theory which acknowledges the difficulty of competing ethical demands may better reflect the complexity inherent in this clinical situation.

Further research into the virtue ethics account of eudaimonia may provide some guidance regarding what medical interventions are striving to achieve, and when they may be hindering rather than helping patients from achieving human flourishing, even at the end of their lives. Further research into patients’ preferences regarding dying and intensive care treatment could provide useful evidence for this discussion and add to the account of what a good death entails.

## Conclusion

This paper has argued that it is not ethically justifiable to provide time-limited trials of intensive care to all critically unwell patients, especially those with a very low chance of surviving their critical illness. Time-limited trials of intensive care treatment have both a small chance of benefiting this patient group and a high chance of harming them by depriving them of a good death. The negative experiences of survivors of intensive care provide an insight into the hidden harms and burdens that such treatments could inflict upon these patients, for the sake of providing as little as a five percent chance of survival.

A deontological ‘rule of rescue’ for the critically unwell fails as an ethical justification of time-limited trials of intensive care. This is because it does not justify time-limiting trials of intensive care treatment, as well as failing to account for the significant experiential costs that intensive care patients face. A consequentialist imperative to achieve survival as the optimal outcome for patients, creating a negative responsibility upon intensive care clinicians for the death of critically unwell patients, also fails as an ethical justification for time-limited trials of intensive care. This is because the ethical imperative to offer time-limited trials of intensive care to all patients, regardless of their chance of survival, overlooks the responsibility of resource-limited intensive care clinicians for suffering caused by their actions.

The consequentialist ethical imperative to optimise the outcome for patients is more effectively realised by employing a patient-specific risk–benefit analysis when deciding whether to offer intensive care treatment. Therefore, the best outcome for patients relies on intensive care clinicians making inherently complex and potentially uncertain decisions, weighing both qualitative and quantitative outcomes. The focus on quantifiable outcomes in clinical applications of consequentialist thinking risks overlooking much of the morally relevant complexity of intensive care decision-making. Consequentialism has not yet provided intensive care clinicians with adequate guidance for how to incorporate complex and uncertain qualitative and quantitative outcomes in the patient-specific risk–benefit analysis.

The importance of the intensive care clinician as a moral agent making decisions in a pressured, complex and uncertain context makes an agent-centred ethical theory, such as virtue ethics, attractive as a source of future ethical resources for intensive care decision-making. The rich concept of human flourishing incorporates both quantitative and qualitative aspects of the human experience and so could provide better guidance for clinical risk–benefit analysis than consequentialism. Further research into the virtue ethics concept of human flourishing, including a good death, has the potential to provide further guidance for intensive care decision-making, grounding it in a fuller understanding of the human experience.

Further research in the form of patient and staff surveys needs to be undertaken to establish the harm-to-benefit ratio that most people would be willing to accept with regards to intensive care treatment. Of particular importance will be elderly and co-morbid patients’ evaluations of their negative intensive care experiences, as these patients are likely to be the ones with the lowest chance of surviving a critical illness. This could then be used to guide intensive care decision-making and to better inform patients of the harms of intensive care treatment. Such research could provide guidance regarding what chance of survival makes the suffering involved in a time-limited trials of intensive care acceptable to patients and ensure that intensive care treatment is not provided to patients with a very low chance of surviving their critical illness, for whom the benefit does not outweigh the cost.
